# Adolescents and young people in sub-Saharan Africa: overcoming challenges and seizing opportunities to achieve HIV epidemic control

**DOI:** 10.3389/fpubh.2024.1321068

**Published:** 2024-03-19

**Authors:** Tafadzwa Dzinamarira, Enos Moyo

**Affiliations:** ^1^School of Health Systems and Public Health, University of Pretoria, Pretoria, South Africa; ^2^ICAP at Columbia University, Lusaka, Zambia; ^3^School of Nursing and Public Health, College of Health Sciences, University of KwaZulu-Natal, Durban, South Africa

**Keywords:** adolescents and young people, prevention, treatment, linkage to care, barriers, strategies

## 1 Introduction

Globally, significant progress has been made in HIV prevention and care. About 76% of people living with HIV (PLHIV) were receiving antiretroviral therapy (ART) in 2022, and 71% of these had viral suppression (1). In the past three decades, having access to ART has prevented about 21 million deaths caused by HIV and AIDS (1). Despite this progress, a substantial number of new infections continue to raise concerns, particularly among adolescents and young people (AYP). About 27% of the 1.3 million new HIV cases worldwide in 2022 occurred in people between the ages of 15 and 24 years. Sixty-six percentage of all new infections among adults aged 15 years and older were among adolescent girls and young women (AGYW) (1). In comparison to older persons, AYP are less likely to have an HIV test, to have a timely link to HIV care, and to remain in care (2).

In sub-Saharan Africa (SSA), AYP are disproportionately affected by HIV due to several factors. Biological, socioeconomic, and cultural factors are some of the contributing factors. As a result of economic marginalization, AYP engage in transactional, age-disparate, and multiple concurrent sexual relationships for survival (3). AYP are also more likely to be engaged in illicit drug use, which put them at risk of HIV transmission (4). Furthermore, AYP face barriers in accessing HIV prevention and treatment services due to being discriminated against at healthcare facilities. Some healthcare workers' bad attitudes toward AYP lead to non-utilization of HIV prevention and treatment services. Particular attention should be paid to AYP who are members of marginalized groups, such as men who have sex with men (MSM), migrant workers, and sex workers, as these groups face heightened risks of HIV infection due to social stigma, discrimination, and limited access to healthcare services. Eba et al. highlighted how AYP who belong to key populations are often stigmatized and discriminated more at healthcare facilities, contributing to a low engagement with HIV prevention and treatment services (5). Since AYP can spread the virus to their older sexual partners, HIV infection among them can have a significant impact on society as a whole. Additionally, AYP who contract HIV and are breastfeeding or pregnant risk passing the disease to their unborn babies.

Comprehensive, high-quality, and integrated HIV prevention and treatment services are necessary to lower HIV transmission among AYP. These services include HIV testing services (HTS), linkage to HIV care and treatment, retention in HIV care and viral suppression, and other support services such as psychosocial support, financial assistance, and legal services (6). Among AYP who are HIV negative, biomedical and non-biomedical HIV prevention services, such as voluntary medical male circumcision (VMMC), provision of condoms, pre-exposure prophylaxis (PrEP), post-exposure prophylaxis (PEP), sexually transmitted infections (STI) screening and treatment, and harm reduction programs are important (7). Leadership and governance, health policy, human resources for health (HRH), capacity building, health information systems, and HIV surveillance are all important factors that can affect the delivery of comprehensive, quality, integrated HIV prevention and treatment services to AYP (8). In this article, we discuss the need for comprehensive, quality, and integrated HIV prevention and treatment services targeting AYP. We discuss barriers and strategies to improve HIV testing services; linkage to HIV care, treatment, and support services; retention in HIV care and viral suppression; and HIV prevention services among AYP.

## 2 HIV testing services

The first of the UNAIDS's 95-95-95 targets to end the HIV epidemic by 2030 is for 95% of PLHIV to know their status (1). HIV testing services are therefore essential for achieving the first 95 (9). HTS include the full spectrum of services that should be provided together with HIV testing. These include pre-test and post-test counseling; linkage to appropriate HIV prevention, treatment, and care services; other clinical and support services; and coordination with laboratory services to promote quality assurance and the delivery of accurate results (10). Numerous countries in SSA have adopted innovative and imaginative HTS strategies, such as facility-based and community-based HTS (11). While voluntary testing and counseling can be provided in communities, in mobile facilities, and outreach facilities, provider-initiated testing and counseling are often facility-based (12). HIV self-testing (HIVST) is a method in which a person tests their blood or saliva for the presence of HIV in a private location at a convenient time (13).

HIV partner service, commonly referred to as disclosure or contact tracing, provides sexual and/or drug-injecting partners of PLHIV with voluntary HTS. HIV partner service is recommended as part of a comprehensive package of HTS and care. Provider-initiated referral and patient referral are two options for this service (10). When a person who has tested positive is directly helped by a trained provider, who contacts their partner or partners and offers them HTS, this is known as a provider-initiated referral. In patient referral, a trained provider advises them to tell their partners they are HIV positive and to obtain HTS (10). Some studies have reported that AYP are less likely than older persons to have an HIV test. Fear of testing, stigma, and discrimination, worries about confidentiality, a lack of services suitable for AYP, insufficient follow-up services, and bad provider attitudes are a few of the reasons that have been mentioned (9, 14). Other obstacles to HIV testing among AYP include perceived low risk of contracting the virus, the perceived psychological burden of having the virus, a lack of privacy at testing facilities, and lengthy waiting times (15).

To improve HTS among AYP, we recommend making testing facilities more accessible. HTS should be located in convenient locations for AYP, such as schools, community centers, and youth centers. They should also be open after school hours to accommodate AYP's schedules. Additionally, testing facilities should ensure privacy and confidentiality, and providers should deliver testing in a friendly and welcoming manner. HTS should also be offered at venues that AYP frequent, such as cinemas, bars, academic institutions, and sporting events. This will make it easier for AYP to get tested without having to go to a traditional medical setting (16). It is also important to strengthen HIV education for communities to actively reduce stigma and discrimination faced by AYP seeking HIV services. Community leaders, including religious leaders, can play a key role in promoting acceptance and understanding of HIV. They can also help to connect AYP with HTS.

Another strategy that can be used to increase the uptake of HTS among AYP is to increase the accessibility of HIVST kits since HIVST was reported to be acceptable among AYP (17, 18). HIVST could increase testing among AYP who shun testing out of fear of phlebotomy or needles (16). HIVST should be delivered via youth-friendly providers and should be combined with peer-delivered education. A study conducted in Malawi and Zimbabwe revealed that HIVST among AYP can be maximized if it is provided through home-based distribution and is affordable (18). A study conducted in Lesotho reported that HIVST gives adolescents greater freedom in terms of how, where, and when they test for HIV, thus improving the uptake of testing coverage, knowledge of HIV status, and linkages to care (17). HIVST and mobile Health (mHealth) solutions may be critical in maintaining progress toward the global 95-95-95 targets among AYP. HIV testing among AYP can be promoted by mHealth interventions that include text messaging, applications, or social media platforms (19). Conditional economic incentives can also be used since they were found to be successful in increasing HIV testing among AYP in Zimbabwe (20).

## 3 Linkage of HIV-positive AYP to treatment and support services

Linkage to care is the process of connecting HIV-positive people to HIV prevention, treatment, and care services. HTS without prompt linkage to treatment services can cause missing or delayed treatment initiation among AYP. Linkage to treatment services usually leads to the screening of comorbid conditions such as tuberculosis, renal impairment, hepatic disorders, anemia, and meningitis. Delayed linkage to treatment services can result in increased morbidity and mortality among AYP (21). Support services that can help PLHIV to manage their health and cope with the challenges of living with HIV include psychosocial support, financial assistance, and legal services (22). Several factors contribute to delayed linkage to treatment and support services among AYP. These factors can be divided into individual, provider, health system, and contextual-level factors (21). Individual factors include fear of stigma and discrimination, denial, fear of disclosing status, and being asymptomatic, while provider-level factors include lack of privacy at healthcare facilities, and the bad attitudes of providers toward AYP (23). Health system factors include the distance to the healthcare facilities, opening hours not conducive to AYP, and waiting times at the facilities (23), while contextual factors include lack of transport costs and poor knowledge about treatment services among AYP (24).

Several strategies that can be used to improve linkage to treatment and support services among AYP who test HIV-positive. One of the strategies is to ensure that HIVST kits have linkage-to-care information. Remote HIVST counseling via telephone or video-chat platforms may allow providers to link AYP to treatment services directly and quickly. After AYP test positive for HIV, providers should follow up with them closely via phone calls and texts to make sure they attend treatment and support services. Financial incentives can also be offered to AYP so that they attend treatment and support services after diagnosis (25).

## 4 Retention in care

The process of keeping PLHIV involved in HIV prevention, treatment, and care programs is known as retention in care. It is crucial since it offers chances to track therapeutic responses, avoid related issues, and offer support services. Retention in care is a necessary prerequisite for ART adherence and viral suppression (26). Poor retention in care increases the risk of poor ART adherence, which increases the chances of drug resistance and treatment failure (27). There are several structural, societal, and healthcare system factors that contribute to the low retention of AYP in care. Structural barriers include unemployment and poverty, whereas social barriers include stigma and discrimination. Distance to medical facilities, ART side effects, and healthcare providers' attitudes are healthcare system factors that influence retention in care among AYP (23).

The retention of care among AYP can be improved by differentiated care models that include devoted weekend clinic time, sexual and reproductive health education, disclosure and ART adherence support, ART refill, individualized peer counseling and support, and peer interaction through sports, art, and games (28). The retention of AYP in care may be improved by community-based ART adherence support groups that meet regularly for group counseling, symptom screening, and the provision of prepackaged ART medicines. mHealth interventions including short message service (SMS)-reminders and check-in messages sent via one-way or two-way SMS have been shown to boost AYP retention in care (29). Economic empowerment of AYP has also been reported to increase retention in care among AYP. This can be offered through training in life skills and financial management. Other empowerment activities include matched financial services for medical expenses, income generation, or education-related expenses (30).

## 5 Linkage of HIV-negative AYP to HIV prevention services

Linking AYP who test HIV-negative to HIV prevention services is crucial since it will help keep them uninfected. Several prevention strategies that exist can be divided into behavioral, biomedical, and structural interventions (7). Behavioral interventions are usually targeted at reducing multiple and concurrent partnerships, erratic condom use, drug abuse, premature sexual debut, and intergenerational sex as all these are drivers of HIV transmission (31). Biomedical interventions include VMMC, PrEP, PEP, STI screening and treatment, and harm reduction programs for illicit drug users (7). Social protection measures include income transfers, career skills training, livelihood training, and micro-credit (32). Access to prevention services by AYP is hampered by several factors such as fear of side effects, stigma and discrimination, low awareness of the services, misconceptions, bad attitudes of healthcare providers, long distances to healthcare facilities, long waiting times at the healthcare facilities, and the cost of the preventive services (33, 34).

To improve the uptake of HIV preventive services among AYP, we recommend that comprehensive HIV education which covers HIV prevention services be reemphasized in schools and colleges so that they have adequate knowledge for decision-making. HIV education should also be provided to communities to reduce stigma and discrimination among AYP accessing HIV prevention services. The HIV education must cover misconceptions about HIV prevention services, as well as the anticipated side effects (35). The preventive services should be offered in youth-friendly clinics by healthcare providers who have a good attitude toward AYP. This will ensure that AYP feel free to access the facilities to receive the services (6). The HIV prevention services should also be offered to AYP at affordable prices since the majority of them in SSA do not have a source of income. Healthcare facilities that offer HIV preventive services should be opened at hours that are convenient for AYP such as weekends and holidays so that the services are accessed more easily by the target group (19). Finally, strengthening community-based initiatives to address misconceptions and concerns surrounding HIV prevention services, particularly regarding potential side effects of medications like PrEP and PEP, will foster open communication and address any lingering doubts that AYP may have.

## 6 Conclusion

The interventions outlined in this paper provide a roadmap for enhancing the uptake of HIV services among AYP and ultimately reducing the burden of HIV in this vulnerable population. To effectively implement the interventions outlined in this paper, strong policy advocacy and development are essential. This includes policy advocacy to secure funding for AYP HIV programs, continuous review of national and regional guidelines for AYP HIV services, capacity building for healthcare providers, community engagement and strengthening data collection and monitoring systems.

## Author contributions

TD: Writing—original draft, Conceptualization. EM: Writing—review & editing, Writing—original draft.
